# Perceived neighbourhood food access is associated with consumption of animal-flesh food, fruits and vegetables among mothers and young children in peri-urban Cambodia

**DOI:** 10.1017/S1368980021004122

**Published:** 2022-03

**Authors:** Minh-Cam Duong, Hung Nguyen-Viet, Delia Grace, Chhay Ty, Huy Sokchea, Vor Sina, Melissa F Young

**Affiliations:** 1 Nutrition and Health Sciences Program, Laney Graduate School, Emory University, Atlanta, GA 30322, USA; 2 Animal and Human Health Program, International Livestock Research Institute, Vietnam and Kenya; 3 Natural Resources Institute, University of Greenwich, London, UK; 4 Livestock Development for Community Livelihood Organization, Cambodia; 5 Hubert Department of Global Health, Emory University

**Keywords:** Neighbourhood food access, Maternal and child nutrition, Urban nutrition, Cambodia

## Abstract

**Objective:**

To examine whether mothers’ perceived neighbourhood food access is associated with their own and their young children’s consumption of animal-flesh food, fruits and vegetables in peri-urban areas of Cambodia.

**Design:**

A cross-sectional survey measured food consumption frequency and perceived neighbourhood food access, the latter including six dimensions of food availability, affordability, convenience, quality, safety and desirability. Multivariate logistic regression was used to assess the association between perceived food access and food consumption.

**Setting:**

Peri-urban districts of Phnom Penh and Siem Reap, Cambodia

**Participants:**

198 mothers of children between 6 and 24 months old.

**Results:**

Over 25 % of the mothers and 40 % of the children had low consumption (< once a day) of either animal-flesh food or fruits and vegetables. Compared with perceived high food access, perceived low food access was associated with an adjusted 5·6-fold and 4·3-fold greater odds of low animal-flesh food consumption among mothers (95 % CI 2·54, 12·46) and children (95 % CI 2·20, 8·60), respectively. Similarly, relative to perceived high food access, perceived low food access was associated with 7·6-times and 5·1-times higher adjusted odds of low fruits and vegetables consumption among mothers (95 % CI 3·22, 18·02) and children (95 % CI 2·69, 9·83), respectively.

**Conclusions:**

Mothers’ perceived neighbourhood food access was an important predictor of their own and their young children’s nutrient-rich food consumption in peri-urban Cambodia. Future work is needed to confirm our findings in other urban settings and examine the role of neighbourhood food environment in the consumption of both nutrient-rich and nutrient-poor food.

Low consumption of nutrient-rich food like animal-flesh food, fruits and vegetables is a major contributing factor to maternal and child undernutrition in low-and-middle-income countries (LMIC). Global policy makers seeking to address undernutrition have called for improving the food environment to promote better dietary quality among mothers and young children^(1,2)^. The food environment refers to the physical, economic, political and socio-cultural surroundings in which consumers engage with the food system to acquire, prepare and consume food^(2)^. Neighbourhoods are the primary space where individuals access and acquire food, which explains the dominant focus of the food environment literature on neighbourhood food access^(3)^.

Among studies of neighbourhood food access, there is a limited attention to undernutrition and inadequate nutrient-rich diet in low-income urban settings. Instead, existing studies have largely focused on the relation between neighbourhood food access and dietary outcomes related to either overnutrition among urban dwellers^(4)^ or undernutrition in rural farming communities^(5)^. The former was carried out in response to the rising incidences of overweight and obesity in LMIC and often examined the over-consumption of fast food, unhealthy snacks and sugar-sweetened beverages^(6–8)^. The latter, appearing in the realm of agricultural economics and focusing mostly on rural Africa, has demonstrated that poor access to fresh food markets is related to low consumption of nutrient-rich food among mothers of reproductive ages and young children between 6 and 24 months of age in rural, farming households^(5)^. These two strands of literature leave a gap in our understanding about neighbourhood food access and inadequate nutrient-rich diet among mothers and young children in low-income urban households, such as those living in peri-urban areas. Peri-urban areas, defined as areas in the peripheral of large cities which undergo a transformation from rural to becoming more urban^(9)^, often experience a high rate of un- or informal employment, unreliable infrastructure and poor public services^(9)^, and thus a high prevalence of underweight, stunting and anemia^(10,11)^. Given the rapid urban expansion in LMIC, it is urgently needed to understand whether and how neighbourhood food access influences nutrient-rich food consumption among low-income, urban dwellers.

This present study aims to contribute to filling this gap by assessing perceived food access rather than the more commonly used measures of objective food access. Perception of food access captures how individuals perceive the availability, affordability, convenience, quality and safety of food in the neighbourhood as well as individuals’ food desirability^(3,12)^. On the other hand, the objective measures usually capture distance to food outlets, characteristics of food outlets and the actual food price. Perceived food access may better reflect the urban food environment, characterised by the co-existence of multiple food sources like supermarkets, wet markets and street vendors^(13)^, because it allows respondents to reflect on their sources of food acquisition rather than having researchers deciding in prior which food outlets to assess. Furthermore, measures of perceived food access can incorporate the dimensions of food quality and safety, which are often omitted from the objective measures. This omission is problematic given that consumers in LMIC are increasingly concerned about food safety and quality^(14,15)^. In addition, previous qualitative studies have highlighted that it is the availability and affordability of safe and good-quality food, and not merely food availability and affordability, that matters to food choice and food acquisition^(16,17)^.

We carried out a pilot study in low-income, peri-urban communities in Cambodia to assess whether mothers’ perceived neighbourhood food access is associated with low consumption of animal-flesh food, fruits and vegetables among themselves and their children between 6 and 24 months of age. Cambodia is a rapidly urbanising Southeast Asian country with a high burden of maternal and child undernutrition^(18)^ and a prevailing concern of food safety^(19)^. Against this backdrop, the current study was conducted as part of the baseline nutrition survey of the Safe Food Fair Food, a programme that aims to develop innovative, market-based interventions to increase food safety in fresh produce markets and ultimately improve nutritional and health outcomes of mothers and young children. The programme focused on children aged between 6 and 24 months since this is the key period of childhood growth and the window of opportunity for food safety and nutrition interventions^(20)^.

## Methods

### Study design and population

We conducted a cross-sectional survey with women who had children between 6 and 24 months of age living in the outskirts of the cities of Phnom Penh and Siem Reap, the two focal provinces of the Safe Food Fair Food program. The survey targeted women living in proximity to the wet markets to provide nutrition baseline data for the subsequent nutrition-sensitive food safety interventions. The survey took place between December 2018 and January 2019, and interviews were administered by trained surveyors with the use of electronic tablets. The electronic data collection was developed using Open Data Kit^(21)^. The data collection was coordinated by a local research organisation, and field workers were trained on the survey questionnaires and the Open Data Kit through several meetings. Oral consent was obtained from participating mothers prior to the enrollment. The work is reviewed by Emory University Institutional Review Board, United States and the National Ethic Committee for Health Research in Cambodia.

Participants were selected using stratified random sampling. In each municipality of Phnom Penh and Siem Reap, we selected seven districts which had the population density between 1500 and 30 000 people/km^2^. We also excluded districts that housed primarily business centres, high-income residential neighbourhoods or homes of government officers. In each district, we selected three communes using systematic sampling and then identified the largest wet market in each commune. Households that lived within 1 km perimeter of the largest wet market and had a child aged 6 to 24 months were recruited by random walk. In random walk, we approached every third household to the right until the sample size was met. Approximately three to five households were selected in each commune, for a total of 100 households in Phnom Penh and 105 in Siem Reap. For each household, one woman was invited for the interviews to provide information about herself and her youngest child who was between 6 and 24 months of age. After the completion of the data collection, seven households were excluded because their children were older than 24 months, resulting in the final analytical sample size of 198 mothers.

### Data collection and measures

We collected information on maternal and household characteristics, sources of food acquisition, food consumption and perceived food access. For each mother, we also asked about food consumption and complementary feeding practice regarding the index child.

#### Socio-demographic characteristics

Demographics variables included self-reported maternal age, number of children, maternal occupation, maternal education, and child age and sex. The socio-economic status was measured by wealth tertiles, which were derived from the first component of the principal component analysis of household assets ownership, an approach commonly used in the Demographic and Health Surveys^(22)^. Food insecurity status was measured using the Household Food Insecurity Access Scale^(23)^ and comprised four categories: food secure, mildly food insecure, moderately food insecure and severely food insecure.

#### Sources of food acquisition

Mothers were asked how frequent (daily, weekly, monthly and never) they acquired animal-flesh food from supermarkets, wet markets, street vendors (i.e. vendors who sell fresh produce outside the designated area of wet markets) and alternative sources. If alternative sources were selected, mothers were asked to specify which alternative sources they used. Similar questions were asked about fruits and vegetables. The choice of these sources was based on an earlier formative research carried out with our study population (unpublished results) and the literature on food acquisition in LMIC^(13)^.

#### Food consumption and dietary diversity

Mothers were also asked how many times they or their children consumed animal-flesh food and consumed fruits and vegetables in a typical week. Low food consumption was defined as consuming the food group less frequent than once a day.

We also collected data on maternal minimum dietary diversity using standard tools developed by FAO and Family Health International 360 (FHI 360)^(24)^ and data on the young and infant child feeding practices using the standard WHO and UNICEF questionnaires^(25)^. In specific, we asked mothers to report which food groups, out of a standard list of food group, mothers consumed during the 24-h period preceding the survey. Similar approach was used to measure children’s dietary diversity.

#### Perceived food access

**Fig. 1:**
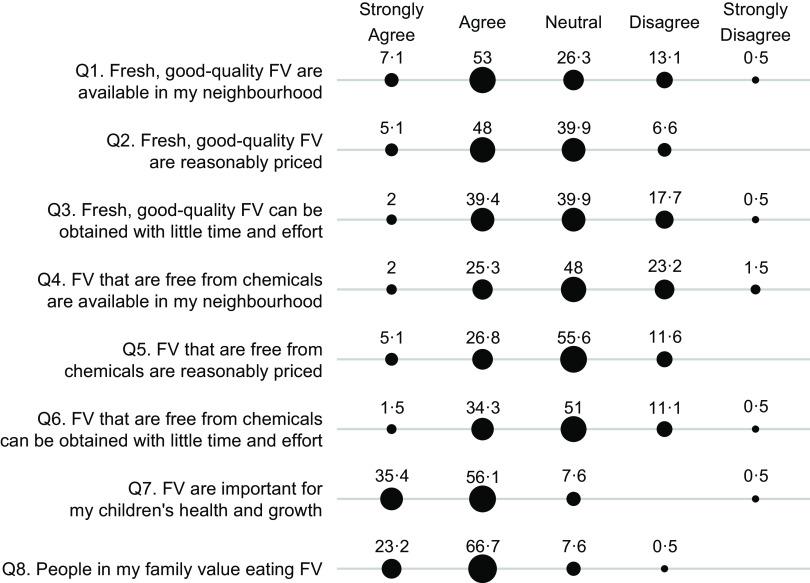
Percentage (%) of participants who responded strongly agree, agree, neutral, disagree or strongly disagree to questions measuring perceived access to fruits and vegetables (FV). Percentage (%) of respondents 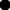, 20; 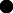, 40; 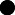, 60

**Fig. 2:**
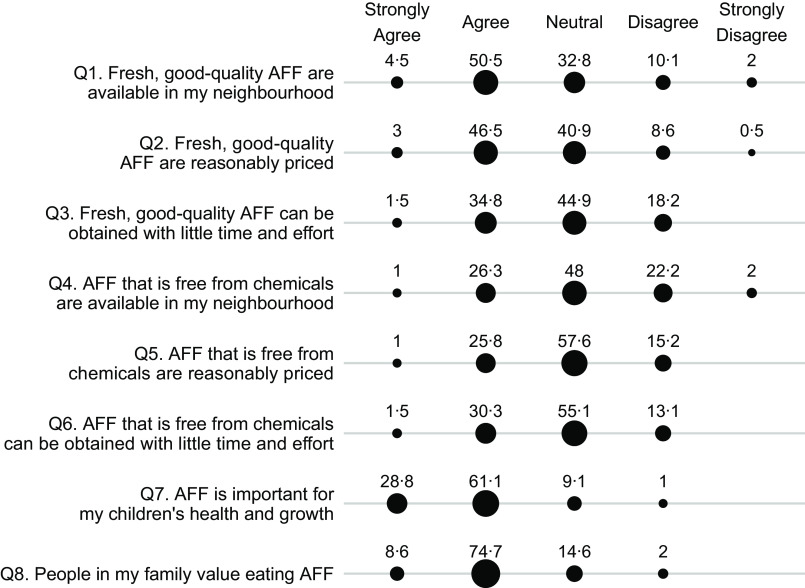
Percentage (%) of participants who responded strongly agree, agree, neutral, disagree or strongly disagree to questions measuring perceived access to animal-flesh food (AFF). Percentage (%) of respondents 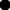, 20; 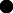, 40; 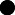, 60

Maternal perceived food access was assessed separately for fruits and vegetables and for animal-flesh food, each through a questionnaire of eight survey items (Figs 1 and 2). The questionnaire was developed to capture six dimensions of food access, namely availability, affordability, convenience, quality, safety and desirability, which were primary dimensions of the food environment^(3,12)^. Additionally, we also worded the survey items to capture the perceived availability and affordability of food that are safe to consume or food that are fresh and of good quality, instead of the availability and affordability of food in general. This design was based on findings from earlier qualitative research, which indicated that consumers did not merely care about proximity and cost of food, but rather the proximity and cost of food that are safe to consume, fresh and of good quality^(16,17)^. In these survey items, food freshness and quality reflected subjective judgement about food attributes such as freshness, taste and nutritional content. On the other hand, food safety referred specifically to perceived chemical contamination rather than perceived biological contamination, because our earlier formative research (unpublished results) and current studies have indicated that contamination of chemicals such as pesticides and additives were the primary food safety concern among consumers^(14,15)^. In all the questions, animal-flesh food is defined as muscle tissue of an animal carcass such as pork, poultry, fish and seafood. We examined perceived food access specifically for animal-flesh food because animal-flesh food like poultry and chicken greatly differs from other animal-sourced food like eggs and milk in terms of food price, the sources of acquisition and perceived food safety risks^(26)^.

The mothers were asked whether they agree or disagree with each item in the five-level Likert scale (1-strongly agree, 2-agree, 3-neutral, 4-disagree or 5-strongly disagree). We calculated Cronbach’s alpha coefficients to assess the internal consistency of the survey items, or the degree to which these items consistently capture the same latent construct^(27)^. We then averaged all the eight survey items to derive a continuous score reflecting the overall perception of access to fruits and vegetables, or to animal-flesh food. Higher scores indicated lower perception of food access. The scores were then dichotomised using the median value as a cut-off. Scores were classified as ‘perceived high food access’ if they were less than or equal the median value and ‘perceived low food access’ if higher than the median value. The Cronbach *α* coefficient is 0·73 for the questionnaire about perceived access to fruits and vegetable and 0·64 for the questions about access to animal-flesh food, indicating an acceptable level of inter-item consistency.

### Statistical analysis

We first described our sample’s socio-demographic characteristics, sources of food acquisition, dietary diversity and food consumption. We reported mean and standard deviation for continuous measures and proportion for categorical measures. We also examined these characteristics by city to understand the variation between the two study sites.

Bivariate and multivariate logistic regressions were used to examine how perceived food access (modelled as a binary predicting variable) were associated with food consumption. We computed the odds of low consumption, defined as less than once a day, as compared with once a day or more. We ran models separately for perceived assess to fruits and vegetables and for perceived assess to animal-flesh food and separately for maternal food consumption and for child food consumption, resulting in a total of four models.

The models examining maternal food consumption were adjusted *a priori* for maternal age, city, wealth tertile and maternal education. In the models related to child food consumption, we adjusted *a priori* for child age and sex, city, wealth tertile and maternal education. All *P*-values < 0·05 were considered significant. We used Variance Inflation Factor and Hosmer–Lemeshow goodness-of-fit test to assess multicollinarity and model fit, respectively.

Nine mothers did not provide answers for one or more perception questions about fruits and vegetables, and three mothers did not answer one or more questions about animal-flesh food. Values for these missing values were estimated with multiple imputation using a Markov Chain Monte Carlos method^(28)^. Ten cycles of imputation were completed to estimate values for the missing covariates. Answers to other perception questions were used in the imputation procedures to predict values of the missing perception questions. We also conducted sensitivity analysis to obtain odd ratios for data without multiple imputation (*n* 189 for models assessing fruits and vegetables consumption, *n* 195 for models assessing animal-flesh food consumption).

All analyses were conducted in R version 3.5.1^(29)^.

## Results

### Respondents’ characteristics

Respondents’ characteristics in the analytical sample and by city are shown in Table 1. Mean age of the mothers and the children was 28·9 (sd = 5·8) years old and 14·6 (sd = 5·1) months old respectively, and over half of the children (59·6 %) were male. Over a third of the mothers did not complete primary school, 32 % were not working and over 50 % experienced some forms of food insecurity. As compared with Phnom Penh, Siem Reap had a higher proportion of women who completed at least primary school (68·7 % *v*. 59·6 %), worked in the agriculture sector (22 % *v*. 0 %), were from the poorest wealth tertile (45·5 % *v*. 21·2 %), and experienced severe food insecurity (21·2 % *v*. 0 %).

**Table 1:** Women’s and child’s socio-demographic characteristics and food consumption by city

	Total (*n* 198)	Phnom Penh (*n* 99)	Siem Reap (*n* 99)	
Characteristics	%	%	%	*P*-value*
**Socio-demographic characteristics**				
Women’s current age				
Mean	28·9	29·3	28·5	0·34
sd	5·8	5·8	5·8	
Child’s age in months				
Mean	14·6	14·5	14·7	0·75
sd	5·1	4·9	5·4	
Child’s sex				
Male	59·6	55·6	63·6	0·31
Female	40·4	44·4	36·4	
No. of children				
1 child	36·9	32·3	41·4	0·06
2 children	38·9	36·4	41·4	
3 or more children	24·2	31·3	17·2	
Maternal education				
None or some primary school	35·9	40·4	31·3	0·006
Completed primary school	21·2	24·2	18·2	
Secondary school	23·7	13·1	34·3	
More than secondary school	19·2	22·2	16·2	
Maternal occupation				
Not working	32·8	37·4	28·3	< 0·001
Agriculture	11·1	0·0	22·2	
Sales and services	38·4	50·5	26·3	
Others†	17·7	12·1	23·2	
Household wealth tertiles				
Poorest	33·3	21·2	45·5	< 0·001
Middle	33·3	28·3	38·4	
Richest	33·3	50·5	16·2	
Household food insecurity status				
Severely food insecure	10·6	0·0	21·2	< 0·001
Moderately food insecure	11·1	5·1	17·2	
Mildly food insecure	35·4	47·5	23·2	
Food secure	42·9	47·5	38·4	
**Sources of food acquisition** ‡				
Fruits and vegetables				
Wet markets	84·3	91·9	76·8	0·006
Street vendors	35·4	37·4	33·3	0·66
Supermarkets	1·5	2·0	1·0	1·00
Alternative (home gardens, rivers, etc.)	26·8	10·1	43·4	< 0·001
Animal-flesh food				
Wet markets	82·8	89·9	75·8	0·01
Street vendors	30·8	27·3	34·3	0·36
Supermarkets	0·5	0·0	1·0	1·00
Alternative (home gardens, rivers, etc.)	14·6	7·1	22·2	0·004
**Food consumption**				
Maternal consumption of fruits and vegetables				
Twice a day or more	62·6	67·7	57·6	0·23
Once a day	11·6	8·1	15·2	
Less frequent than once a day	25·8	24·2	27·3	
Do not consume	0·0	0·0	0·0	
Maternal consumption of animal-flesh food				
Twice a day or more	68·7	79·8	57·6	< 0·001
Once a day	5·1	1·0	9·1	
Less frequent than once a day	26·3	19·2	33·3	
Do not consume	0·0	0·0	0·0	
Child consumption of fruits and vegetables				
Twice a day or more	35·4	31·3	39·4	0·22
Once a day	18·7	17·2	20·2	
Less frequent than once a day	35·9	37·4	34·3	
Do not consume	10·1	14·1	6·1	
Child consumption of animal-flesh food				
Twice a day or more	45·5	45·5	45·5	0·003
Once a day	12·6	5·1	20·2	
Less frequent than once a day	33·8	37·4	30·3	
Do not consume	8·1	12·1	4·0	
**Dietary diversity & child feeding**				
Maternal dietary diversity				
Minimum dietary diversity	44·9	48·5	41·4	0·39
Below minimum dietary diversity	55·1	51·5	58·6	
Child’s dietary diversity				
Minimum dietary diversity	47·0	41·4	52·5	0·15
Below minimum dietary diversity	53·0	58·6	47·5	
Children ever breastfed				
Yes	89·9	83·8	96·0	0·008
No	10·1	16·2	4·0	
Initiation of breast-feeding§				
Immediately	32·2	31·7	32·6	1·00
Not immediately	67·8	68·3	67·4	
Initiation of complementary food between 6 and 8 months				
Yes	79·8	78·8	80·8	0·86
No	20·2	21·2	19·2	

* *P*-value is extracted from *t* test for age and the Fisher-exact test for other variables.

† Other occupations include professionals, skilled manuals and unskilled manuals.

‡ This section presents the percentage of women who acquired food from each source on a daily or weekly basis. The sum exceeds 100 % since many women reported acquiring food from more than one sources. Thus, the Fisher-exact tests were carried out for each individual source of food acquisition.

§ There were twenty-one missing values in the data on initiation of breast-feeding.

Most mothers reported acquiring food from multiple sources. Over 80 % of the mothers purchased animal-flesh food, fruits and vegetables daily or weekly from wet markets and over 30 % purchased from street vendors on the daily or weekly basis. In addition, about 26 % of the mothers procured fruits and vegetables and 14 % acquired animal-flesh food from alternative sources, including growing food in the home gardens and catching fish from rivers. As compared with Siem Reap, Phnom Penh had a higher proportion of mothers who acquired fruits and vegetables from wet markets (91·9 % *v*. 76·8 %) and a lower proportion who acquired fruits and vegetables from the alternative sources (10·1 % *v*. 43·4 %). Similar results were found for animal-flesh food. In both cities, purchase from supermarkets was rare.

About 25·8 % and 26·3 % of the mothers reported low consumption of fruits and vegetables and low consumption of animal-flesh food (less frequent than once a day), respectively. In children, 35·9 % consumed fruits and vegetables less than once a day and 10·1 % did not consume at all while 33·8 % consumed animal-flesh food less frequent than once a day and 8·1 % did not consume this food group at all. There were no significant differences in fruits and vegetables consumption by city of residence. However, Siem Reap had a higher proportion of mothers with low animal-flesh food consumption (33·3 % *v*. 19·2 %). In contrast, Phnom Penh had a larger share of children who had low consumption (37·4 % *v*. 30·3 %) or did not consume animal-flesh food (12·1 % *v*. 4·0).

Less than half of the mothers and children met the minimum dietary diversity. About 90 % of the children were ever breastfed, about a third were immediately breastfed and around 80 % started solid food between 6 and 8 months.

### Perceived food access survey items

When asked about perceived access to fruits and vegetables (Fig. 1), more mothers agreed with the statements about food quality and freshness (Q1–Q3) than with the statements about food safety (Q4–Q6). For example, when asked about neighbourhood availability of fresh, good-quality fruits and vegetables (Q1), 60·1 % agreed/strongly agreed, 26·3 % responded neutral and 13·6 % disagreed/strongly disagreed. On the other hand, when asked about neighbourhood availability of chemical-free fruits and vegetables (Q4), 27·3 % agreed/strongly agreed, 48 % responded neutral and 24·7 % disagreed/strongly disagreed. Lastly, over 90 % agreed or strongly agreed with the statements about the importance and the value of eating fruits and vegetables (Q7–Q8).

Questions about animal-flesh food (Fig. 2) also yielded similar responses: more mothers agreed with the statements about food quality and freshness (Q1–Q3) than with the statements about food safety (Q4–Q6). For example, 55 % agreed/strongly agreed with the statement about the availability of good-quality animal-flesh food (Q1) as compared with 27·3 % who agreed/strongly agreed with the statement about the availability of chemical-free animal-flesh food (Q4). With regard to the importance and values of eating animal-flesh food (Q7–Q8), over 80 % responded agreed or strongly agreed.

### Multivariate analysis

In the multivariate analysis of maternal food consumption (Table 2), perceived low access to fruits and vegetables in the neighbourhood was associated with 7·61 times greater odds of low fruits and vegetables consumption (95 % CI 3·22, 18·02) compared with perceived high access. Similarly, mothers having low perception of access to animal-flesh food were 5·63 times more likely to report low animal-flesh food consumption (95 % CI 2·54, 12·46) than mothers having high perception. Household wealth was associated with the consumption of animal-flesh food but not with fruit and vegetable consumption.

**Table 2:** Logistic regression analysis of the characteristics associated with women’s low consumption of fruits and vegetables and animal-flesh food

	Low fruits and vegetable consumption (< once a day)						Low animal-flesh food consumption (< once a day)					
	Univariate analysis (*n* 198)			Multivariate analysis (*n* 198)*			Univariate analysis (*n* 198)			Multivariate analysis (*n* 198)*		
	OR	95 % CI	*P* value	OR	95 % CI	*P* value	OR	95 % CI	*P* value	OR	95 % CI	*P*-value
Maternal age	0·96	0·91, 1·02	0·18	0·96	0·89, 1·02	0·19	1·00	0·95, 1·06	0·99	1·01	0·95, 1·08	0·68
City of residence												
Phnom Penh	ref			ref			ref			ref		
Siem Reap	1·17	0·62, 2·22	0·63	1·34	0·61, 2·99	0·47	2·11	1·10, 4·04	0·025	1·96	0·86, 4·46	0·11
Wealth tertile												
High	ref			ref			ref			ref		
Middle	2·25	1·00, 5·05	0·05	2·15	0·81, 5·69	0·13	5·71	2·15, 15·19	< 0·001	3·40	1·14, 10·17	0·028
Low	1·56	0·68, 3·60	0·30	1·12	0·39, 3·22	0·84	5·00	1·87, 13·36	0·001	4·10	1·25, 13·42	0·020
Maternal education												
More than secondary education	ref			ref			ref			ref		
Secondary education	3·25	0·96, 10·98	0·06	1·70	0·43, 6·70	0·45	7·64	1·61, 36·16	0·010	5·00	0·94, 26·45	0·06
Completed primary education	2·32	0·65, 8·27	0·19	1·14	0·27, 4·77	0·86	9·00	1·89, 42·90	0·006	7·31	1·36, 39·39	0·021
None or some primary education	4·62	1·47, 14·51	0·009	3·53	0·94, 13·28	0·06	8·08	1·79, 36·59	0·007	4·11	0·79, 21·45	0·09
Access to fruits & vegetables†,‡												
Perceived high access	ref			ref								
Perceived low access	7·37	3·24, 16·77	< 0·001	7·61	3·22, 18·02	< 0·001						
Access to animal-flesh food†,§												
Perceived high access							ref			ref		
Perceived low access							4·49	2·23, 9·03	< 0·001	5·63	2·54, 12·46	< 0·001

ref, reference category.

* The multivariate model includes maternal age, city of residence, wealth tertile, maternal education and either score on access to fruits and vegetables or score on access to animal-flesh food.

† Missing data for perceived food access were imputed with multiple imputation using Markov Chain Monte Carlos method.

‡ Mothers were classified as having perceived low access if their average perception score was higher than 2·375 and perceived high access if otherwise.

§ Mothers were classified as having perceived low access if their average perception score was higher than 2·5 and perceived high access if otherwise.

Similar results were found in multivariate models of child food consumption (Table 3). Compared to perceived high access, perceived low access to fruits and vegetables was associated with 5·14 times greater odds of low fruits and vegetables consumption in children (95 % CI 2·69, 9·83). There was also significant association between perceived low access to animal-flesh food and children’s low animal-flesh food consumption (OR 4·34; 95 % CI 2·20, 8·60). Children who were younger, born to mothers with lower education attainment or lived in Phnom Penh were more like to have low animal-flesh food consumption. Household wealth was not associated with fruits and vegetables consumption, nor with consumption of animal-flesh food among children.

**Table 3:** Logistic regression analysis of the characteristics associated with children’s low consumption of fruits and vegetables and animal-flesh food

	Low fruits and vegetable consumption (< once a day)						Low animal-flesh food consumption (< once a day)					
	Univariate analysis (*n* 198)			Multivariate analysis (*n* 198)*			Univariate analysis (*n* 198)			Multivariate analysis (*n* 198)*		
	OR	95 % CI	*P* value	OR	95 % CI	*P* value	OR	95 % CI	*P* value	OR	95 % CI	*P*-value
Child age	0·96	0·91, 1·10	0·12	0·94	0·88, 1·00	0·07	0·92	0·87, 0·98	0·006	0·91	0·85, 0·97	0·005
Child sex												
Male	ref			ref			ref			ref		
Female	0·94	0·53, 1·66	0·82	0·87	0·45, 1·66	0·66	0·88	0·49, 1·56	0·65	1·03	0·52, 2·04	0·93
City of residence												
Phnom Penh	ref			ref			ref			ref		
Siem Reap	0·64	0·36, 1·12	0·12	0·57	0·28, 1·17	0·12	0·53	0·30, 0·95	0·031	0·45	0·22, 0·95	0·035
Wealth tertile												
High	ref			ref			ref			ref		
Middle	1·36	0·68, 2·69	0·38	1·60	0·67, 3·84	0·30	1·28	0·64, 2·56	0·48	1·04	0·42, 2·58	0·93
Low	0·94	0·47, 1·87	0·86	1·08	0·44, 2·66	0·86	1·07	0·53, 2·14	0·86	1·13	0·44, 2·88	0·80
Maternal education												
More than secondary education	ref			ref			ref			ref		
Secondary education	3·18	1·27, 8·00	0·014	2·57	0·88, 7·52	0·08	5·33	1·77, 16·05	0·003	6·30	1·79, 22·21	0·004
Completed primary education	2·80	1·09, 7·18	0·032	1·72	0·59, 5·03	0·33	9·71	3·15, 29·88	< 0·001	11·63	3·26, 41·49	< 0·001
None or some primary education	2·72	1·15, 6·43	0·022	1·90	0·68, 5·28	0·22	5·42	1·89, 15·48	0·002	4·58	1·38, 15·21	0·013
Access to fruits and vegetables†,‡												
Perceived high access	ref			ref								
Perceived low access	5·07	2·75, 9·35	< 0·001	5·14	2·69, 9·83	< 0·001						
Access to animal-flesh food†,§												
Perceived high access							ref			ref		
Perceived low access							3·63	2·00, 6·57	< 0·001	4·34	2·20, 8·60	< 0·001

ref, reference category.

* The multivariate model includes child age, child sex, city of residence, wealth tertile, maternal education and either score on access to fruits and vegetables or score on access to animal-flesh food.

† Missing data for perceived food access were imputed with multiple imputation using Markov Chain Monte Carlos method.

‡ Mothers were classified as having perceived low access if the average perception score is more than 2·375 and ‘perceived high access’ if otherwise.

§ Mothers were classified as having perceived low access if the average perception score is more than 2·5 and ‘perceived high access’ if otherwise.

In all models, there was no evidence of multicollinarity between independent variables (GVIF < 2 in all models) and no evidence of poor fit according to Hosmer and Lemeshow goodness of fit test (*P*-value > 0·05). Multivariate analysis without imputation yielded similar results (see online supplementary material, Supplemental Table 1).

## Discussion

We present a novel evidence about the relation between perceived neighbourhood food access and nutrient-rich food consumption in low-income, peri-urban areas in Cambodia. Our results indicate that after adjusting for socio-demographic characteristics, mothers having low perception of food access were more likely to have low consumption (less than once a day) of animal-flesh food, fruits and vegetables. Similarly, their low perception of food access was also related to low consumption of animal-flesh food, fruits and vegetables among their children between 6 and 24 months of age.

Our findings confirm the previously found association between perceived food access and fruits and vegetables consumption, although our findings were drawn from mothers and young children while most of the prior work were carried out with adults and adolescences^(30–35)^. Among these studies, several reported significant associations^(30–32)^ while others showed null findings^(33–35)^. A reason for the mixed results is the use of different instruments to operationalise perceived food access. Most instruments captured the dimensions of food availability^(30–35)^ and food quality^(30,31,33,35)^ but only a few integrated affordability^(30,32)^, convenience (i.e. ease of access)^(32,34)^ or food safety. Mujahid et al.^(36)^ have validated a four-item perception instrument, but the instrument is more relevant to cardiovascular outcomes and lacks affordability, convenience and food safety dimensions. These dimensions matter in the context of urban LMIC. In our study, 24·7 % of the respondents disagreed that fruits and vegetables free from harmful chemicals were available in the neighbourhood and 11·6 % disagreed that this food group was affordable. A mixed-method study in Myanmar has indicated that consumers perceived food quality and healthfulness through the lens of food safety^(37)^, and another study in Mexico showed that perceived food quality influenced the decisions where, how much and how often to acquire food^(16)^. To account for the interactions between these different dimensions, our perception questions asked about the availability, affordability and convenience of acquiring good-quality and safe food instead of the general food. Future work can adopt similar approach to develop and validate a perception scale of food access relevant to urban LMIC. Such a validated scale is needed to enable comparisons across studies and countries.

Our study contributes to the literature of urban nutrition by showing the correlation between perceived low food access and low animal-flesh food consumption in urban mothers and young children. One study conducted in urban Ethiopia also indicated the association between perceived affordability, but not perceived availability, with household consumption of meat and eggs^(38)^. Prior studies about rural farming communities have also found similar associations, although they often used physical measures of food access like distance and travel time to food markets^(5)^. A systematic review of these studies has indicated that the strength of the associations varied by context and depended on other factors like nutritional knowledge and farm production^(5)^. In our study, the magnitude of the associations is large; for example, perceived low food access was associated with 5·63 times (95 % CI 2·54, 12·46) and 4·34 times (95 % CI 2·20, 8·60) higher odds of low animal-flesh food consumption among mothers and children, respectively. In the study of Ethiopian urban dwellers, the odd ratios relating perceived food access and food consumption were also moderate to high, ranging between 1·7 and 5·4. Future work is needed to confirm the direction and the strength of the association between neighbourhood food access and dietary quality among urban mothers and young children.

It is important to point out that our participants lived within 1 km of a large wet market and yet had diverting perceptions about food access, which supports the hypothesis that better proximity to food markets does not guarantee more favourable perceived food access^(39)^. This hypothesis can explain why interventions that improved neighbourhood food availability alone yielded inconsistent effects on food purchase and consumption^(40)^ but showed positive results if combined with other components like food affordability and desirability^(41,42)^. Theoretical and empirical studies of food choice have also indicated that food purchase and consumption decisions are complex, contextual and driven by inter-related factors^(43,44)^. Building on these evidence, our findings support the multi-component approach in food environment interventions, where multiple aspects are taken into account to influence the overall perception of neighbourhood food access, which can lead to changes in food purchase^(45)^, home food availability^(46)^ and ultimately food consumption.

One unexpected finding in our study is that household wealth is not related to the consumption of animal-flesh food among children, although we found the association with mothers’ animal-flesh food consumption. Prior work has found a household wealth gradient in both mothers’ and children’ consumption of animal-sourced food including eggs, dairy and animal-flesh food, but these studies drew evidence from nationally or sub-nationally representative data, where there was a large variation in household wealth^(47–49)^. Our study participants resided in peri-urban neighbourhoods locating close to a wet market, which might lead to higher homogeneity in wealth and thus the lack of association with child food consumption. It is possible that diet of young children between 6 and 24 months, as compared with mothers’ diet, is less sensitive to changes in the household wealth and instead more sensitive to other factors such as neighbourhood food access, as demonstrated in our findings, or food norms and beliefs. Focus groups discussions with Ethiopian^(50)^ and Chinese^(51)^ mothers both indicated that mothers did not feed their young children meat or vegetables because of the belief that children lacking teeth cannot chew and digest these food. Interventions seeking to promote better child diet through income-generating activities, such as livestock management or micro-financing, should also address other factors such as food norms or neighbourhood food access.

Our study has several limitations. First, due to the cross-sectional nature of the data, it is not possible to ascertain whether improvements in maternal perceived food access would lead to more frequent consumption of nutrient-rich foods. Second, while our instruments measuring perceived food access yielded acceptable internal consistency, they have not been validated elsewhere. Lastly, we sampled households living in the proximity to the wet markets and thus are not able to generalise our findings to a more general urban population with varying distance to food markets. Further research is needed to expand this work to other urban contexts.

## Conclusion

Our study provides evidence that in low-income urban settings mothers’ perceived access to food in their neighbourhood is associated with their own and their young children’s consumption of animal-flesh food, fruits and vegetables. These findings suggest that neighbourhood food access might play a role in the consumption of nutrient-rich foods and thus potentially nutrient adequacy of diet and the risk of micronutrient deficiencies among women and children. We additionally showed the heterogeneity of the urban food environment in Cambodia, where wet markets, street vendors and alternative sources like home gardens and wild food environment were important sources of fresh produce. These aspects of the urban food environment should be adequately assessed in future programmes and policies that aim to improve food access and nutrition in Cambodia. Future research is needed to expand our findings to examine the role of perceived neighbourhood food environment in addressing the dual burden of undernutrition and overnutrition in LMIC.

## Supplementary material

For supplementary material accompanying this paper visit https://doi.org/10.1017/S1368980021004122. click here to view supplementary material
