# Supplementary material for: Perceived neighbourhood food access is associated with consumption of animal-flesh food, fruits and vegetables among mothers and young children in peri-urban Cambodia
Source: Public Health Nutr. 2021 Oct 1;25(3):717–28. doi: 10.1017/S1368980021004122 (PMC9991642; doi:10.1017/S1368980021004122)
Supplement: Supplementary file 1 [file S1368980021004122sup001.pdf]

Supplementary table 1: Multivariate logistics regression* analysis of the characteristics associated with low consumption (less than once a day) of fruits and vegetables and animal-flesh food in mothers and children without multiple imputation of the perception variables.

	**Maternal food consumption (< once a day)**				**Child food consumption (< once a day)**			
	Fruits & vegetables (n=189)		Animal-flesh food (n=195)		Fruits & vegetables (n=189)		Animal-flesh food (n=195)	
	OR (95% CI)	*P* value	OR (95% CI)	*P* value	OR (95% CI)	*P* value	OR (95% CI)	*P* value
Maternal age	0.95 (0.89 – 1.02)	0.13	1.01 (0.95 – 1.08)	0.74				
Child age					0.94 (0.88 – 1.00)	0.06	0.91 (0.85 – 0.97)	0.005
Child sex								
Male					ref		ref	
Female					0.94 (0.48 – 1.83)	0.85	1.01 (0.51 – 2.00)	0.97
City of residence								
Phnom Penh	ref		ref		ref		ref	
Siem Riep	1.12 (0.49 – 2.55)	0.79	1.98 (0.87 – 4.49)	0.10	0.53 (0.25 – 1.10)	0.09	0.47 (0.22 – 0.98)	0.04
Wealth tertile								
High	ref		ref		ref		ref	
Middle	2.69 (0.98 – 7.42)	0.06	3.22 (1.08 – 9.67)	0.037	1.73 (0.69 – 4.34)	0.24	1.12 (0.45 – 2.81)	0.80
Low	1.27 (0.43 – 3.74)	0.67	3.88 (1.18 – 12.77)	0.025	1.10 (0.44 – 2.79)	0.84	1.23 (0.48 – 3.17)	0.67
Maternal education								
More than secondary education	ref		ref		ref		ref	
Completed or some secondary education	1.75 (0.44 – 7.02)	0.43	4.99 (0.94 – 26.32)	0.06	2.86 (0.95 – 8.60)	0.06	5.95 (1.70 – 20.86)	0.005
Completed primary education	1.06 (0.25 – 4.51)	0.94	4.41 (1.38 – 39.80)	0.020	1.69 (0.57 – 5.03)	0.35	10.55 (2.96 – 37.62)	<0.001
None or some primary education	3.57 (0.95 – 13.49)	0.06	4.34 (0.83 – 22.67)	0.08	1.89 (0.67 – 5.33)	0.23	4.35 (1.31 – 14.48)	0.017
Perceived access to fruits and vegetables†								
Satisfied	ref				ref			
Dissatisfied	8.25 (3.42 – 19.90)	<0.001			5.54 (2.83 – 10.85)	<0.001		
Perceived access to animal-flesh food‡								
Satisfied			ref				ref	
Dissatisfied			5.48 (2.48 – 12.12)	<0.001			4.25 (2.14 – 8.42)	<0.001

ref, reference category
* All variables entered into the multivariated models were listed in the table.
† Mothers were classified as having perceived low access if their average perception score was higher than 2.375 and perceived high access if otherwise
‡ Mothers were classified as having perceived low access if their average perception score was higher than 2.5 and perceived high access if otherwise
